# The Efficacy of New Zealand Greenshell™ Mussel Powder Supplementation in Supporting Muscle Recovery Following Eccentric Exercise-Induced Muscle Damage in Healthy, Untrained Adult Males

**DOI:** 10.3390/nu15102316

**Published:** 2023-05-15

**Authors:** Dominic Lomiwes, Matthew Barnes, Odette Shaw, Nayer Ngametua, Greg Sawyer, Natalie Burr, Duncan Hedderley, Alexander Kanon, Tracey Bear, Andrew Carroll, Kerry Bentley-Hewitt, Hong Sabrina Tian, Matthew R. Miller

**Affiliations:** 1 The New Zealand Institute for Plant and Food Research Limited, Nutrition and Health Group, Food Innovation, Palmerston North 4410, New Zealand; odette.shaw@plantandfood.co.nz (O.S.); nayer.ngametua@plantandfood.co.nz (N.N.); greg.sawyer@plantandfood.co.nz (G.S.); natalie.burr@plantandfood.co.nz (N.B.); duncan.hedderley@plantandfood.co.nz (D.H.); tracey.bear@plantandfood.co.nz (T.B.); andrew.carroll@plantandfood.co.nz (A.C.); kerry.bentley-hewitt@plantandfood.co.nz (K.B.-H.); 2 School of Sport, Exercise and Nutrition, Massey University, Palmerston North 4410, New Zealand; m.barnes@massey.ac.nz; 3 School of Food and Advanced Technology, Massey University, Auckland 0632, New Zealand; h.tian@massey.ac.nz; 4 Cawthron Institute, Nelson 7010, New Zealand; matt.miller@cawthron.org.nz

**Keywords:** Greenshell™ mussel, green-lipped mussel, muscle damage, muscle recovery, inflammation, delayed onset muscle soreness

## Abstract

Unaccustomed eccentric exercise results in muscle damage limiting physical performance for several days. This study investigated if Greenshell™ mussel (GSM) powder consumption expedited muscle recovery from eccentric exercise-induced muscle damage (EIMD). Methods: Twenty untrained adult men were recruited into a double-blind, placebo-controlled, cross-over study and were randomly assigned to receive the GSM powder or placebo treatment first. Participants consumed their allocated intervention for four weeks then completed a bench-stepping exercise that induced muscle damage to the eccentrically exercised leg. Muscle function, soreness and biomarkers of muscle damage, oxidative stress and inflammation were measured before exercise, immediately after exercise and 24, 48 and 72 h post exercise. GSM powder promoted muscle function recovery, significantly improving (*p* < 0.05) isometric and concentric peak torque at 48 h and 72 h post exercise, respectively. Participants on the GSM treatment had faster dissipation of soreness, with significant treatment × time interactions for affective (*p* = 0.007) and Visual Analogue Scale-assessed pain (*p* = 0.018). At 72 h, plasma creatine kinase concentrations in the GSM group were lower (*p* < 0.05) compared with the placebo group. This study provides evidence for GSM powder being effective in supporting muscle recovery from EIMD.

## 1. Introduction

Unaccustomed and prolonged eccentric exercise leads to muscle damage, resulting in reduced muscle strength and mobility [1,2], delayed onset of muscle soreness (DOMS) [3] and elevated levels of circulating muscle-related proteins [1] that persist for several days. Exercise-induced muscle damage (EIMD) effects on muscle function and soreness significantly affect exercise performance and impede the execution of daily physical tasks [4].

While the mechanisms underpinning EIMD and associated symptoms such as DOMS are not precisely known, reduced muscle function from muscle damage is attributed to the disturbance of the highly ordered sarcomere structure of skeletal muscle [5]. Muscle damage may attenuate the release of Ca^2+^ from the sarcoplasmic reticulum to the cytosol, thereby reducing the binding of myofibrillar filaments, which drive muscle contraction [6]. Immune cells, such as macrophages and neutrophils, can contribute to additional inflammatory damage as they infiltrate the injury site to clear damaged muscle tissue [7].

There is increasing demand for nutritional strategies to reduce the severity of muscle damage or to expedite muscle recovery from EIMD. A variety of plant- and marine-derived foods to support muscle recovery following EIMD have been researched, and variable efficacy of polyphenol-rich foods and marine oils in various formats in accelerating the recovery of muscle performance and reducing DOMS following EIMD has been reported [8,9,10].

New Zealand green-lipped mussel (*Perna canaliculus*), also known as Greenshell™ mussel (GSM), is rich in bioactive lipids, polysaccharides and peptides [11,12]. These bivalve molluscs are endemic to New Zealand and are the country’s most significant marine export product where they are commercially farmed for food and nutraceutical products. GSM has been traditionally recognised as a dietary staple and prepared or preserved for consumption using various methods including steaming, curing, dehydrating and fermentation (see review by Miller et al. [13]). Although the health benefits of consuming GSM have been traditionally acknowledged, recent research has highlighted its potential to support various health outcomes and mitigate disease. Clinical studies have demonstrated the efficacy of GSM supplements in improving arthritis and asthma symptoms in [14,15] and improving cognitive outcomes in children and adolescents with high levels of inattention, hyperactivity and impulsivity [16]. Moreover, several intervention trials have also investigated the benefits of GSM oil supplementation in supporting muscle recovery from EIMD [17,18,19]. These studies showed that long-term GSM oil supplementation was effective in reducing loss in muscle function and attenuating DOMS, inflammation and biomarkers of muscle damage following eccentric EIMD.

The benefits of GSM oil in supporting muscle recovery from EIMD have been attributed to the anti-inflammatory properties of bioactive constituents including in the ω-3 polyunsaturated fatty acids (PUFAs) (eicosapentaenoic acid (EPA) and docosahexaenoic acid (DHA)), sterols, sterol esters and polar lipids [20]. GSM oil has been previously demonstrated to attenuate arachidonic acid metabolism to inflammatory eicosanoids and leukotrienes by dually inhibiting cyclooxygenase-2 and lipoxygenase enzyme activities post exercise [21].

Although there is compelling clinical evidence supporting the efficacy of GSM oil for muscle recovery following EIMD, it is unclear whether supplementation with GSM powder is equally beneficial. PernaUltra™ is a flash-dried powder of GSM whole meat, prepared using a standardised proprietary manufacturing process. It is a complex blend of bioactive lipids, polysaccharides and proteins [22], which may synergistically support muscle recovery following EIMD. The primary objective of this study was to investigate the effect of consuming PernaUltra GSM powder on markers of muscle damage following eccentric exercise-induced damage to the quadriceps. We hypothesised that supplementation of GSM powder would expedite muscle recovery compared with the placebo.

## 2. Materials and Methods

### 2.1. Study Participants

A total of 22 healthy male volunteers between 21 and 45 years old who were not undergoing any form of exercise training and had similar fitness characteristics based on the Baecke habitual questionnaire [23] were enrolled in this study. Volunteers were excluded from the study if their respective Baecke habitual activity and sports scores were above 3 and 3.5 or had known allergies to mussels or mussel-derived products. All recruited participants completed a health screening questionnaire and confirmed that they did not have any medical conditions or injury that may be exacerbated by the exercise requirements of this study. All recruited participants signed an informed consent form. This study’s methods and procedures were reviewed and approved by New Zealand’s Southern Health and Disability Ethics Committee (20/STH/75) and registered at the Australian New Zealand Clinical Trials Registry (ACTRN12620000565943).

### 2.2. Study Design

This was a randomised, double-blind, placebo-controlled, cross-over intervention study. Eligible participants attended a familiarisation session at least one week prior to commencing the study. In this session, they were instructed on how to perform the bench-stepping exercise that they would be required to complete on their exercise trial day and the muscle function assessments on an isokinetic dynamometer (Biodex Medical Systems Inc., New York, NY, USA) that they would be required to undergo during their exercise trial and recovery days.

All participants underwent a six-week dietary washout period where they omitted foods containing New Zealand GSM and supplements and foods high in omega-3 fatty acids eicosapentaenoic acid (EPA) and docosahexaenoic acid (DHA). Participants were randomised into either the PernaUltra GSM powder or placebo intervention group for the study’s first arm and asked to consume their allocated intervention daily for four weeks. Participants arrived on location within 24 h of their final supplementation day to complete their exercise trial day.

Participants avoided strenuous exercise 48 h prior to their scheduled trial day. Upon arrival at the laboratory, participants completed the short-form McGill pain questionnaire (SF-MPQ) [24] to rate the present pain of the leg assigned for eccentric exercise. After venipuncture blood donation, participants completed a five-minute warmup on a Cyclus2 cycle ergometer (RBM elektronik-automation GmbH, Leipzig, Germany) at a work rate of 100 W followed by measurement of pre-exercise muscle function in both legs. For these tests, participants performed five maximal eccentric, isometric and concentric quadricep contractions and five maximal concentric hamstring contractions while seated on a Biodex isokinetic dynamometer. Subjective scores of perceived soreness of the exercising leg were collected during the first eccentric and isometric contraction using a visual analogue scale (VAS). Participants then completed six five-minute bouts of the bench-stepping protocol to induce muscle damage to the quadriceps of the eccentric exercised leg. Participants then rated the present resting pain of their eccentrically exercised leg on the SF-MPQ and then donated a second venous blood sample. After donating the second venous blood sample, post-exercise quadriceps muscle function of the eccentrically and non-eccentrically exercised leg was assessed on the isokinetic dynamometer.

Participants returned to the facility 24, 48 and 72 h post exercise for recovery assessments. On each visit, participants completed the SF-MPQ before donating a venous blood sample. After completing a five-minute warmup on a cycle ergometer, muscle function was assessed with the Biodex isokinetic dynamometer as well as perceived pain during the first eccentric and isometric contractions. During the 72 h recovery period, participants avoided strenuous exercise and continued to omit New Zealand GSM and foods and supplements that contained high concentrations of EPA and DHA from their diet.

**Figure 1:**
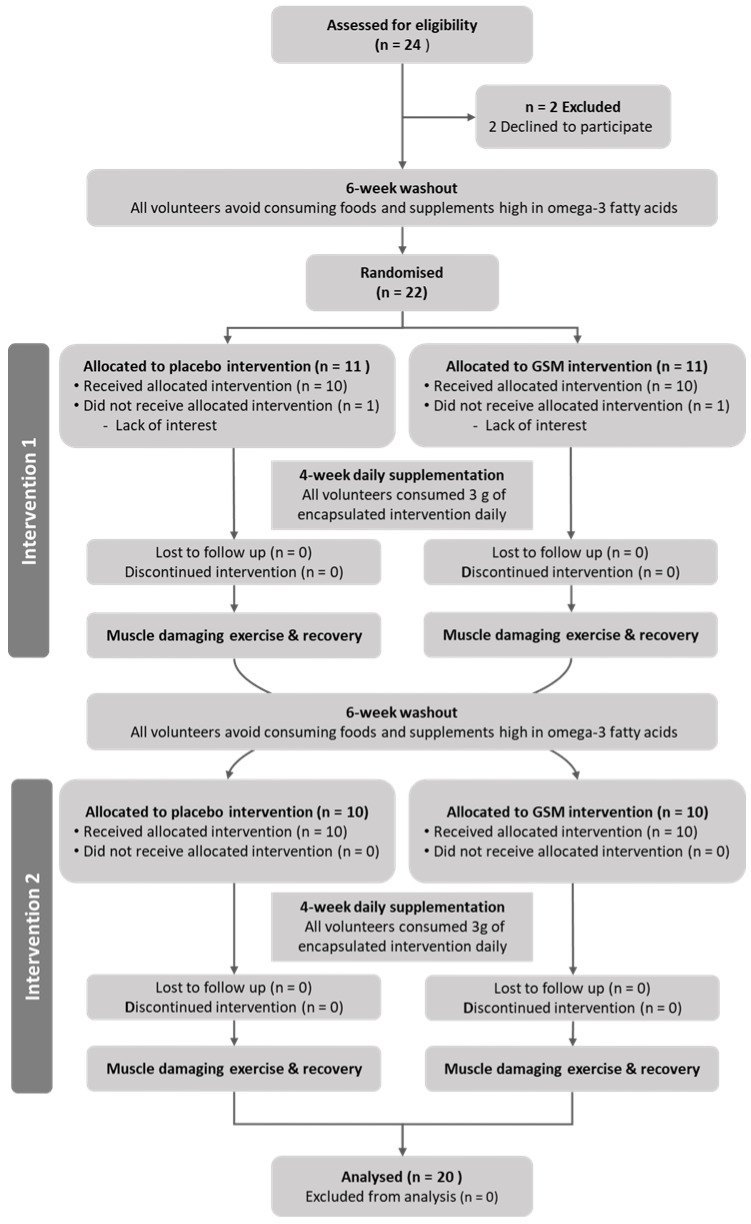
Consolidated Standards of Reporting Trials (CONSORT) diagram illustrating the flow of participants through each stage of the randomised crossover trial.

Before the beginning of the second trial arm, participants underwent another six-week dietary washout period. In the second trial arm, participants were supplemented with the dietary intervention that they were not allocated to during the first trial arm. Participants underwent the same muscle damage and recovery protocols but with the opposite leg to the one that had undergone eccentric muscle damage during the first trial arm. The participants’ progress through the phases of this randomised, cross-over study is illustrated in the Consolidated Standards of Reporting Trials (CONSORT) flow diagram (Figure 1).

### 2.3. Dietary Intervention

The GSM and placebo interventions used in this study and the methods to characterise their composition have previously been described [25,26]. Briefly, flash-dried whole meat GSM powder was produced by Sanford Ltd. (Pernaultra, Enzaq facility, Blenheim, New Zealand). Sunflower seed protein (BP Bulk powders, Braeside, Melbourne, VIC, Australia) was selected as a placebo as it is relatively similar to GSM powder in respect to macronutrient composition and is a neutral source of non-bioactive protein. Both GSM and placebo powders were encapsulated with the same opaque gelatine capsules with each capsule containing 0.5 g of powder. Once encapsulated, the GSM and placebo capsules were indistinguishable to trial coordinators and participants. Participants were required to consume a total of 3 g (six capsules) of their allocated intervention daily for the duration of the supplementation period, which is equivalent to 1–2 mussels. To track the participants’ compliance to the supplementation regimen, they were given the week’s allocated capsules in a seven-day compartment pill box at the beginning of each week. At the end of each week, the participants returned any capsules that they did not consume and received the following week’s capsules.

### 2.4. Exercise Protocol

The bench-stepping exercise protocol used in this study was modified from Newham et al. [1]. This protocol has been demonstrated to induce measurable muscle damage to the quadriceps of the eccentrically exercised leg. Briefly, participants completed six five-minute bouts of bench stepping at a cadence of 15 steps per minute, with each bout separated by two minutes of passive rest. The step height was adjusted to 110% of participants’ lower leg length (measured length of knee joint to heel). During the stepping exercise, the quadriceps of the leading leg concentrically contracted while stepping up and stepping down with the same leg ensuring that the quadriceps of the contralateral leg contracted eccentrically. Participants maintained a consistent pace during the exercise to ensure maximal tension during each concentric and eccentric movement. All participants performed the stepping exercise while wearing a vest containing an additional weight equivalent to 15% of their bodyweight.

### 2.5. Muscle Function Assessment

Participants were seated on a Biodex isokinetic dynamometer at a position where the femoral epicondyle of the tested leg was aligned with the machine’s axis of rotation. The ankle strap of the machine was positioned 5 cm proximal to the test leg’s medial malleolus, and the ankle and thigh of the test leg were strapped firmly to the machine to isolate the movement of the quadriceps. The range of motion of the test leg was set at 60° for concentric and eccentric contractions and fixed at 75° for isometric contractions using the dynamometer’s goniometer. Participants performed five maximal eccentric, isometric and concentric contractions with each contraction type separated by one minute of passive rest. Participants maintained five seconds of maximal effort during each isometric contraction. The torque during concentric contractions was measured at an angular velocity of 30°/s. The absolute peak torque from the five contractions for each movement was recorded.

### 2.6. Subjective Pain Assessments

Perceived muscle soreness during muscle function assessments was determined during the exercise trial and recovery (24, 48 and 72 h post exercise) days. Participants rated the severity of soreness of the exercising leg during the first maximal eccentric and isometric contractions on a 5-point Likert scale ranging from 1 to 5 (1 = no soreness; 5 = worse possible pain).

The muscle soreness of the resting eccentrically exercised leg was assessed with the SF-MPQ before the bench-stepping exercise and at selected post-exercise timepoints (0, 24, 48 and 72 h post exercise). The questionnaire comprised 15 pain-related adjectives, and participants rated each adjective on scale of 0 (none) to 3 (severe). The sum from each adjective score was calculated to give the total pain measure. The pain descriptors in the SF-MPQ included 4 affective pain and 11 sensory pain adjectives, allowing for the specific assessment at these 2 pain dimensions. In addition, the SF-MPQ included a VAS scale anchored by descriptors “no pain” and “worse possible pain” at the opposite ends of the 10 cm scale to assess average pain experienced by participants.

### 2.7. Blood Sampling

Venous blood samples were collected into heparin and ethylenediaminetetraacetic acid (EDTA) vacutainer tubes and centrifuged at room temperature. The plasma layer and erythrocytes were collected, aliquoted and stored at −80 °C until measurement of creatine kinase (CK), cartilage oligomeric matrix protein (COMP), oxidative stress, antioxidant capacity and cytokine concentrations.

For isolation of peripheral blood mononuclear cells (PBMC), the buffy coat interface containing mononuclear cells following centrifugation was collected and washed with Dulbecco’s phosphate-buffered saline (DPBS). Following incubation in erythrocyte lysis buffer (Sigma; Cat. No. R7757, St. Louis, MO, USA), cells were washed and suspended in DPBS for subsequent cell staining.

### 2.8. Creatine Kinase

Plasma CK collected pre and immediately post exercise and 24, 48 and 72 h post exercise was measured at a commercial laboratory (Canterbury Health Laboratories, Christchurch, New Zealand). The analysis of CK was quantified by spectrophotometry, which measured the rate of nicotinamide adenine dinucleotide (NADH) formation from an enzymatic reaction using an automated Beckman Coulter AU5822 analyser. Plasma, and not serum, was the preferred analyte by the laboratory, and the reference intervals for plasma CK in males were reported to be between 60 and 22 U/L.

### 2.9. Immune Measures

#### 2.9.1. Plasma Cytokine

Plasma cytokine concentrations were analysed by bead array using Biolegend Legendplex™ 13-plex Human Essential Response panel (Cat. No. 740930, San Diego, CA, USA) according to the manufacturer’s instructions. The simultaneous quantitation of 13 cytokines (interleukin (IL)-4, IL-2, C-X-C motif chemokine ligand (CXCL)10 (IP-10), IL-1β, tumour necrosis factor (TNF)-α, monocyte chemoattractant protein (MCP)-1, IL-17A, IL-6, IL-10, interferon (IFN)-γ, IL-12p70, CXCL8 (IL-8), and transforming growth factor (TGF)-β1) in plasma samples was measured using a BD FACSverse™ flow cytometer (BD Biosciences, San Jose, CA, USA).

#### 2.9.2. Immune Cell Phenotyping

Isolated PBMC were first stained with Biolegend “zombie NIR” fixable viability dye (Cat. No. 423106; San Diego, CA, USA) according to the manufacturer’s instructions for 15 min at room temperature. Cells were washed in DPBS before being stained with fluorescently labelled cell surface markers at 4 °C for 15 min. The Biolegend antibodies used were PE-Fire700 CD45 (Haematopoietic cell marker); BV605 CD3ε (T cells/natural killer T (NKT) cells); BV510 CD4 (T helper/Treg); BV650 CD8a (Cytotoxic T cells/NKT cells); BV711 CD25 (Treg/activated T cells); APC-Fire750 CD127 (Treg); PerCP CD38 (Activated NKT cells/Activated T cells/B cells); BV421 CD68 (Myeloid cells); BV570 CD56 (NK cells/NKT cells/T cells); PE-Cy7 CD16 (natural killer (NK) cells/monocytes/neutrophils); AF488 CD14 (Monocytes/neutrophils); PE-Cy5 CD15 (Neutrophils); AF647 CD11b (monocytes/macrophages); PE CD11c (monocytes/dendritic cells); BV750 HLA-DR (activated monocytes/DCs). The PBMCs were fixed in 4% formalin and then suspended in fluorescence-activated single cell sorting (FACS) buffer and analysed on a Cytek Aurora Spectral 3 laser flow cytometer (Cytek Biosciences, Fremont, CA, USA) with live spectral unmixing. Cell phenotype subpopulations were identified from Flow Cytometry Standard (FCS) files using the FlowJo software (BD Biosciences, San Jose, CA, USA).

### 2.10. Statistical Analysis

Statistical analysis of data was conducted using analysis of variance (ANOVA), based on linear mixed effect models with fixed effects for trial arm, treatment and time × treatment interaction and random effects for participant, participant × trial arm and participant × time. Residuals were inspected to check that the assumptions of the model were satisfied; for many of the antioxidant and cytokine measures, data were log-transformed before analysis to stabilise the variance. For most data, raw means are presented; for antioxidant and cytokine measures, there were a few missing samples which influenced the raw data means. For these measures, predicted means were calculated, and where the data had been log-transformed, the predicted means were back-transformed. Models were fitted with the R package Imer Test, and post-hoc tests were performed using the predict means package.

## 3. Results

### 3.1. Physical Characteristics of Participants

The variation in age, height and weight of participants recruited in this study is presented in Table 1. Moderate Work and Sports index scores from the Baecke questionnaire confirm that all volunteers were physically untrained. The low variation in these indices also indicate that participants had very similar habitual activity.

Anthropometric measures of each volunteer were measured using manual scales, and habitual activity was determined using the Baecke Physical Activity Questionnaire. Vest weight worn by participants was equivalent to 15% of their bodyweight, and the bench height was set at 110% of participants’ lower leg length. Data are mean ± SD.

### 3.2. Muscle Function

Eccentric exercise from bench-stepping exercise induced a significant decline (time effect; *p* ≤ 0.001) in the peak isometric torque of the quadriceps in both groups that did not recover to pre-exercise values following 72 h post exercise. While no significant treatment effect was measured for this parameter (*p* = 0.368), a significant treatment × time interaction was detected (*p* = 0.014). Pairwise comparisons revealed significantly higher quadriceps isometric torque in the GSM group compared with the placebo group 48 h and 72 h post exercise.

**Figure 2:**
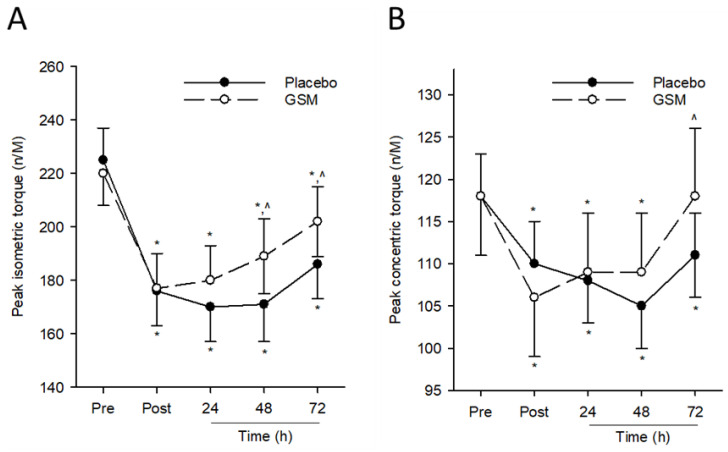
Muscle function assessments after bench-stepping exercise. Peak quadriceps isometric (**A**) and hamstring concentric torque (**B**) were assessed before exercise (Pre) and 0 (Post), 24, 48 and 72 h after bench stepping in eccentrically exercised quadriceps. * indicates significant difference from pre-exercise scores (*p* < 0.05). ^ indicates significant difference from placebo (*p* < 0.05). GSM, Greenshell™ mussel.

For peak concentric torque, there was a significant reduction (time effect; *p* ≤ 0.001) in this parameter in both groups following repeated eccentric contractions from bench stepping (Figure 2B). While there was no treatment effect for this parameter (*p* = 0.105), there was a significant treatment × time interaction (*p* = 0.012). Pairwise comparisons between treatment groups revealed significantly higher peak concentric torque in the GSM group compared with the placebo group 72 h post exercise. Peak concentric torque in the GSM group, but not the placebo group, were similar to pre-exercise values. Taken together, these results indicate the efficacy of GSM supplementation in expediting the recovery of muscle function following an acute bout of eccentric exercise.

### 3.3. Subjective Pain Assessments

**Figure 3:**
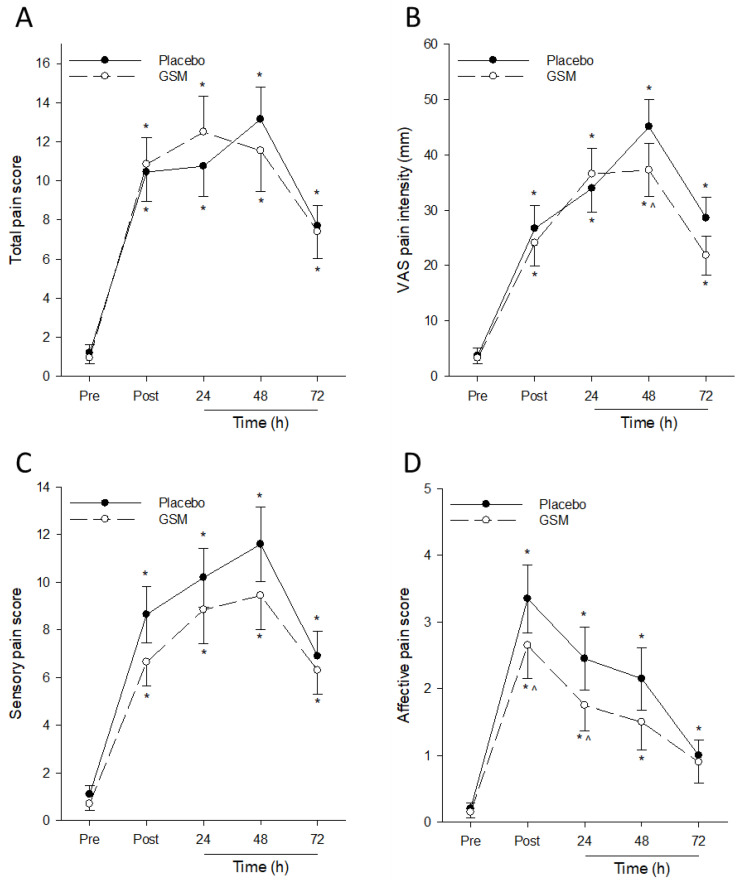
Pain assessment of eccentrically exercised leg at rest after bench-stepping exercise. Total (**A**), Visual Analogue Scale (VAS)-assessed (**B**), sensory (**C**) and affective (**D**) pain measures were collected before exercise (Pre) and 0 (Post), 24, 48 and 72 h after bench stepping following 4 weeks of supplementation with the placebo or Greenshell™ mussel (GSM) intervention. Data are means ± SEM. * indicates significant difference from baseline measure (*p* < 0.05). ^ indicates significant difference from placebo at corresponding timepoint (*p* < 0.05).

Muscle damage from repeated eccentric contractions is typically accompanied by DOMS that persists for several days after exercise. The post-exercise sensory, affective, total and VAS-assessed pain of the eccentrically exercised leg at rest significantly increased (*p* < 0.001) from pre-exercise values in both GSM and placebo groups (Figure 3). There was a significant treatment × time (*p* = 0.018) interaction for VAS-assessed pain score, which significantly increased post exercise, peaking at 48 h post exercise in both treatment groups (Figure 3B). Pairwise comparisons at 48 h post exercise revealed significantly lower average pain in the GSM group compared with the placebo group, indicating reduced severity of pain experienced by those in the GSM group following EIMD.

Total pain scores of the resting eccentrically exercised leg significantly increased in both treatment groups post exercise (time effect; *p* < 0.001) and remained significantly above pre-exercise values at 72 h post exercise in both treatment groups (Figure 3A). When the SF-MPQ questionnaire was stratified into the sensory and affective pain subscales, a significant treatment effect (*p* = 0.007) was detected for affective pain (Figure 3D). Affective pain scores significantly increased post exercise, peaking immediately after bench stepping and declining thereafter over the 72 h recovery period in both treatment groups. Pairwise comparisons between treatment groups revealed that affective pain in the GSM group was significantly lower compared with the placebo group immediately after and 24 h post exercise (*p* < 0.05). In comparison, no significant treatment (*p* = 0.091) and treatment × time (*p* = 0.358) interactions were observed for sensory pain (Figure 3C). Taken together, reductions in peak affective pain and VAS pain following eccentric exercise indicate GSM is effective at reducing the severity of DOMS that accompanies EIMD.

**Figure 4:**
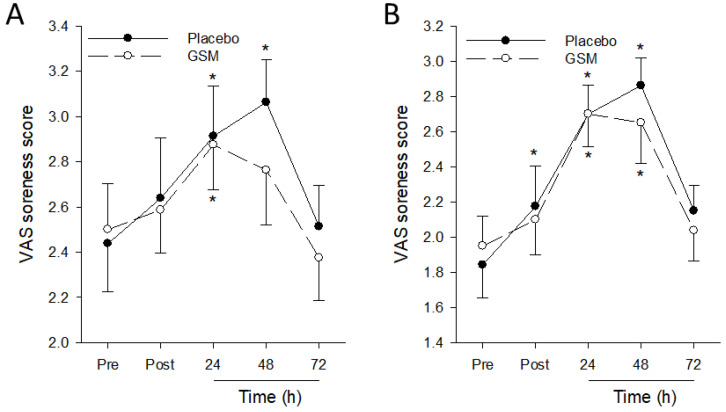
Muscle soreness scores during eccentric (**A**) and isometric (**B**) maximal voluntary contractions of eccentrically exercised quadriceps before (Pre) and 0 (Post), 24, 48 and 72 h after the bench-stepping exercise following 4 weeks of supplementation with placebo or Greenshell™ mussel (GSM) intervention. Data are means ± SEM. * indicates significant difference from pre-exercise scores (*p* < 0.05). VAS, Visual Analogue Scale.

When muscle soreness during maximal voluntary contractions was assessed, a significant (*p* = 0.015) time effect was observed for muscle soreness in the eccentrically exercised leg during maximal eccentric contractions, but no treatment (*p* = 0.836) and treatment × time effects (*p* = 0.674) were detected (Figure 4). Soreness scores during maximal eccentric and isometric contractions significantly increased from pre-exercise scores, peaking at 24 h or 48 h post exercise, then declining thereafter. Muscle soreness scores during eccentric contractions in the GSM group returned to pre-exercise scores 48 h post exercise when soreness scores during this movement in the placebo group were at their post-exercise peak, indicating the effect of GSM supplementation in reducing the severity of muscle soreness during exercise following EIMD.

### 3.4. Creatine Kinase

**Figure 5:**
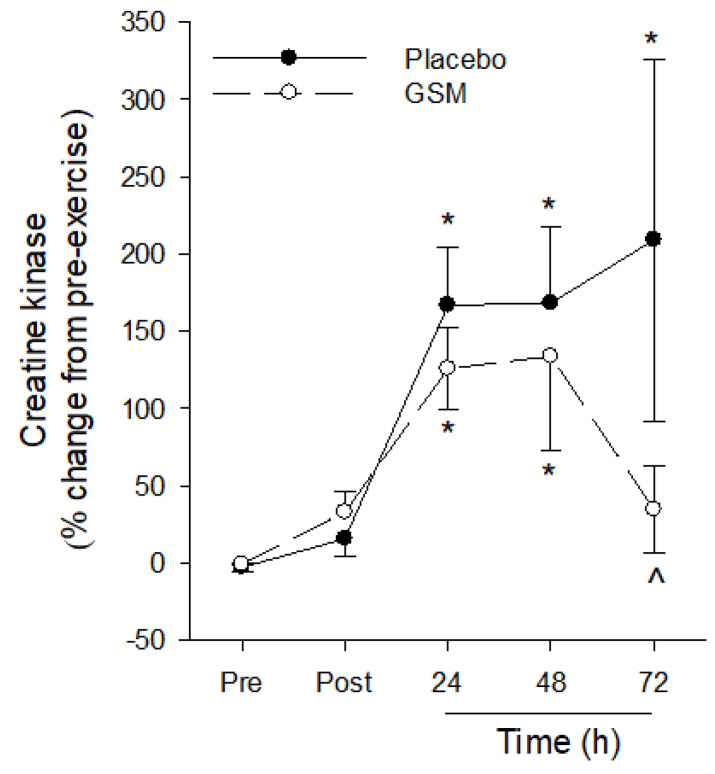
Plasma creatine kinase concentrations pre-exercise (Pre) and 0 (Post), 24, 48 and 72 h post exercise following 4 weeks of placebo or Greenshell™ mussel (GSM) supplementation. Data are means ± SEM and expressed as % change from pre-exercise concentrations. * indicates significant difference from baseline concentration (*p* < 0.05). ^ indicates significant difference from placebo at corresponding timepoint (*p* < 0.05).

The bench-stepping exercise induced a significant change in plasma CK (time effect: *p* < 0.001) with plasma CK concentrations significantly higher 24 h and 48 h post exercise compared with pre-exercise concentrations in both GSM and placebo groups (Figure 5). At 72 h, plasma CK in the placebo group remained significantly higher compared with pre-exercise concentrations. In comparison, plasma CK in the GSM group returned to pre-exercise concentrations and was significantly lower (*p* < 0.05) compared with the placebo group at this timepoint.

### 3.5. Immune Measures

**Figure 6:**
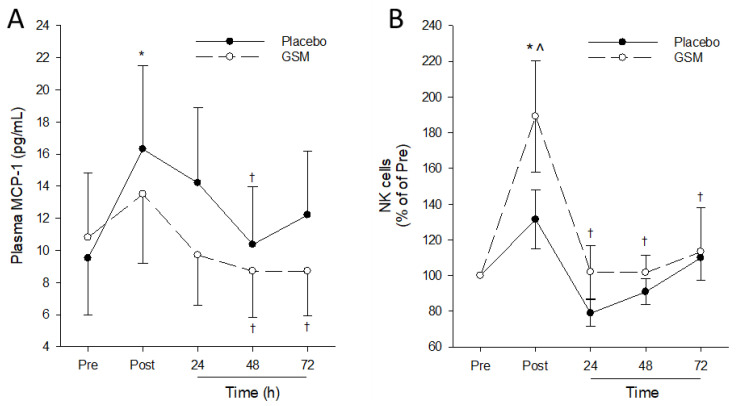
Plasma monocyte chemoattractant (MCP-1) concentration (**A**) and relative circulating natural killer (NK) cell counts (**B**) pre-exercise (Pre) and 0 (Post), 24, 48 and 72 h after the bench-stepping exercise. Data are means ± SEM. * denotes significant difference (*p* < 0.05) from Pre values. † denotes significant difference (*p* < 0.05) from Post values. GSM, Greenshell™ mussel. ^ indicates significant difference from placebo (*p* < 0.05).

A significant time effect (*p* = 0.009) was measured for plasma MCP-1 concentrations, but no treatment (*p* = 0.292) and time × treatment (*p* = 0.154) effects were detected. Pairwise comparisons within the treatment group revealed a significant increase (*p* < 0.05) in plasma MCP-1 immediately after the bench-stepping exercise in the placebo group that declined to pre-exercise concentrations by 24 h post exercise so that MCP-1 was significantly lower at 48 h post exercise compared with concentrations measured immediately post exercise (Figure 6A). After the exercise, the GSM group exhibited a small increase in plasma MCP-1, which was not statistically significant (*p* > 0.05). The MCP-1 concentrations then declined and were significantly lower at 48 h and 72 h compared with the concentrations measured immediately post exercise. Bench-stepping exercise did not induce significant increases in any of the remaining 12 cytokines post exercise (Table S1). While plasma concentrations of selected cytokines declined during the recovery period, no significant treatment differences were detected (*p* > 0.05).

No time, treatment or time × treatment interactions were measured for circulating total and sub populations of T cells, monocyte and neutrophils, indicating that exercise and GSM supplementation had no effect on the circulating populations of these immune cells post exercise (Figure S1). There was a significant time effect for circulating NK cells (*p* < 0.001) (Figure 6B). While exercise did not exert any significant changes in circulating NK cell counts in the placebo group, a significant increase (*p* < 0.05) in circulating NK cells was observed in the GSM group immediately post exercise. This declined to pre-exercise counts following 24 h, 48 h and 72 h recovery, with significantly lower (*p* < 0.05) NK cell counts at these timepoints compared with post-exercise values. Pairwise comparisons between treatment groups confirmed that post-exercise NK cell counts in the GSM were significantly higher compared with the placebo group.

## 4. Discussion

The current study investigated the effectiveness of daily supplementation with GSM powder for four weeks to accelerate muscle recovery after EIMD in untrained males. Our findings show that GSM powder supplementation expedited muscle recovery following exercise-induced damage as determined by faster recovery of muscle function, reduction in plasma CK concentrations and decreased soreness of target muscle groups. The potential mechanism of action for GSM powder in supporting muscle recovery may be partially through modulating the post-exercise immune responses to facilitate muscle repair. Together, these results support our hypothesis that consuming GSM powder supports muscle recovery in untrained males following EIMD to the quadriceps. To our knowledge, this is the first study demonstrating the benefits of GSM powder as a functional food for supporting muscle recovery after exercise.

Reduced muscle function following prolonged unaccustomed eccentric exercise is a classic indicator of muscle damage. Previous studies using a bench-stepping paradigm have reported up to a 20% reduction in peak isometric torque that persisted for 48 h post exercise in untrained cohorts [1,27]. Similarly, our results showed reductions in peak isometric (23%) and concentric (22%) torque of the quadriceps and hamstrings, respectively, in the placebo group that did not return to pre-exercise levels even after 72 h of recovery. However, the duration of reduced muscle function following EIMD was minimised in the GSM group as indicated by the faster recovery of peak isometric and concentric torque during recovery compared with when participants consumed the placebo.

Previous nutrition intervention studies have investigated the benefits of supplementing with GSM and other marine-derived lipids in attenuating eccentric EIMD. Using a downhill running protocol to induce muscle damage to the quadriceps, supplementation with a GSM-derived lipid blend did not attenuate exercised-induced reductions in peak concentric and isometric torque of the quadriceps in trained and untrained runners [17,28]. However, supplementation with GSM lipid extract was found to protect from EIMD reductions in knee joint range of motion in untrained males [17,18]. Inconsistencies on the efficacy of GSM-derived supplements in supporting muscle function recovery following EIMD may be attributed to differences in the exercise models utilised. Declines in muscle function following bench stepping are generally greater compared with downhill running [29]. We used a bench-stepping protocol in this study as it may serve as a more sensitive model for detecting the benefits of nutritional interventions in expediting muscle recovery following EIMD.

Delayed onset muscle soreness (DOMS) is a common outcome of unaccustomed physical activity, typically occurring between 24 to 72 h after exercise and gradually subsiding thereafter. The discomfort caused by DOMS adversely affects the execution of daily activities and athletic performance [4]. Using the SF-MPQ to assess DOMS in the resting eccentrically exercised leg, our findings are consistent with previous research indicating that bench stepping significantly increased muscle soreness, peaking at 48 h post exercise [30,31]. Previous studies have shown that supplementation with GSM oil reduces the severity of DOMS following downhill running in both trained [19] and untrained [18] men and accelerates the dissipation of DOMS to pre-exercise levels within 48 h post exercise [17]. Similarly, our study found that GSM supplementation reduced peak VAS-assessed pain 48 h post exercise. Pain is a bi-dimensional experience that exists at a sensory (intensity) and affective (unpleasantness) dimension [32]. While no treatment effects were observed for post-exercise total and sensory pain, GSM powder supplementation attenuated peak affective pain and accelerated the resolution of affective pain to pre-exercise levels. Taken together, our findings suggest the positive effects of GSM powder in alleviating post-exercise DOMS by reducing the perceived unpleasantness of DOMS rather than its intensity.

Eccentric exercise can disrupt the highly arranged structure of skeletal muscle, releasing CK into circulation, which is a recognised biomarker of EIMD. Bench-stepping exercise has been reported to induce a delayed increase in plasma CK, peaking at 48 to 72 h post exercise and returning to baseline concentrations after 7 days of recovery [1,30,31]. Our results showed a significant increase in plasma CK 24 h post exercise in both GSM and placebo groups, confirming that muscle damage was induced by the bench-stepping protocol. Previous studies have reported the efficacy of GSM-derived supplements in reducing muscle damage, as indicated by circulating CK, in untrained cohorts. Supplementation with GSM-derived oils attenuated [17] or completely mitigated [18] post-exercise increases in circulating CK in untrained men following downhill running, which also corresponded with reduced severity of DOMS. Our study revealed a trend towards lower concentrations of post-exercise CK and significantly lower CK concentrations in the GSM group at the later recovery timepoints. Collectively, long-term GSM powder supplementation expedited the recovery of muscle function, attenuated the severity of DOMS and hastened the dissipation of soreness and circulating CK, which provides evidence for the efficacy of GSM powder in supporting muscle recovery following EIMD.

The functional benefits of GSM supplementation for supporting muscle recovery are largely attributed to the anti-inflammatory bioactivity of GSM constituents. Exercise-induced muscle damage signals the increased release of cytokines by skeletal muscle and resident immune cells, resulting in an acute dynamic inflammatory response. Of the cytokines measured in this study, only MCP-1 was significantly upregulated after exercise, and the significant increase in MCP-1 observed immediately post exercise was mitigated in the GSM powder group. MCP-1 is a potent chemokine that regulates the migration and infiltration of monocytes into damaged muscle tissue, where they differentiate to macrophages and clear damaged myofibers, facilitating the remodelling and repair of damaged muscle [33]. Eccentric exercise has been reported to increase plasma MCP-1, which has been associated with post-exercise monocytosis [34,35]. Additionally, increases in MCP-1 gene and protein expression in skeletal muscle following eccentric muscle damage have been associated with the increased infiltration of macrophages in skeletal muscle [36,37]. While no changes in the post-exercise population of classical CD16-CD14+ monocytes in both intervention groups were detected in the present study, this is likely due to the lack of measurements at earlier post-exercise timepoints (6 h) when monocytosis has been reported to peak [34,35].

Muscle damage protocols, such as repeated eccentric quadriceps maximal voluntary contractions and bench stepping, induce an increase in IL-6 that peaks 6 h post exercise then returns to pre-exercise concentrations following 24 h recovery [29,38]. Muscle damage to the quadriceps from downhill running has also been shown to induce TNF-α and IL-6 concentrations post exercise, and the protracted elevation of these cytokines was significantly reduced by supplementation with GSM and fish oil blends [10,17,18]. Given the absence of measures at critical post-exercise timepoints where inflammatory cytokines and key immune cell populations have been demonstrated to peak, it is difficult to infer whether the benefits of GSM powder supplementation in supporting muscle recovery are mediated by the immune modulatory properties of this product.

An interesting finding in this study was the intervention effect on post-exercise circulating NK cell populations. Acute exercise has been shown to lead to an immediate increase in circulating NK cell numbers post exercise that returns to pre-exercise levels within 24 h of recovery [39]. Additionally, exercise training has been shown to increase NK cell number, which may be beneficial for providing greater immune surveillance and protection from infection [40]. Findings from this study showed a significant increase in circulating NK cells immediately post exercise in the GSM group but not the placebo group. Although their role in facilitating muscle recovery is unclear, NK cells potentially regulate post-exercise muscle repair by mediating the infiltration of monocytes and neutrophils to damaged muscle to facilitate the muscle remodelling and repair [41]. Further research is needed to clearly define the utility of an augmented circulating NK cell population post exercise in expediting muscle recovery following EIMD.

Previous intervention studies have postulated that the anti-inflammatory and analgesic effects of GSM may only be partially explained by the bioactive properties of EPA and DHA [17]. This is because the total dose of ω-3 PUFAs used in these studies was lower than the daily dose of EPA and DHA previously shown to reduce EIMD [9,10]. Given that the daily dose of PUFAs consumed in this study was lower compared with GSM oil intervention studies, it is plausible that other bioactive lipid and non-lipid constituents may act in synergy with EPA and DHA to promote muscle recovery. Hyaluronic acid, a glycosaminoglycan, has been shown to reverse inflammation-mediated inhibition of muscle stem cells to emerge from quiescence and initiate muscle repair in vitro [42]. Other potential GSM bioactives include phospholipids (i.e., phosphatidylcholine and phosphatidylserine) [43] and novel GSM-derived peptides which have been demonstrated to support muscle recovery and exert in vitro anti-inflammatory bioactivity [44]. Further studies are required to characterise the bioavailability of these GSM constituents and their bioactivity to promote muscle recovery from exercise.

It is important to acknowledge that the present study had limitations. Participants were solely untrained men; therefore, the findings in this study may not be extrapolated to women or trained individuals. Training status has a significant influence on the physiological and biological parameters of muscle damage due to the highly adaptable nature of skeletal muscle to repeated exercise [19,37]. Untrained participants were recruited to maximise the effect of the bench-stepping protocol on parameters of muscle damage, thereby maximising the potential of detecting GSM effects on these measures. Despite the GSM-related changes in post-exercise plasma MCP-1 and circulating NK cell population, further evidence is required to confirm the bioactivity of GSM powder in modulating the immune response following EIMD as previous exercise studies have reported. This limitation is due to the absence of measurements at critical time points early post exercise where key inflammatory cytokines have been demonstrated to temporally peak.

## 5. Conclusions

The present study demonstrates that long-term supplementation with New Zealand GSM powder expedited the recovery of muscle function, reduced the severity of DOMS and facilitated the dissipation of DOMS and CK to baseline during recovery following EIMD to the quadriceps. These findings support the potential benefits of GSM powder as a functional food for supporting muscle recovery in untrained men. Further research is required to investigate whether GSM confer similar benefits in untrained women and trained athletes. Future research to characterise whether the efficacy of GSM powder in supporting muscle recovery is mediated by the immune-modulatory properties of GSM powder constituents is recommended.

## Figures and Tables

**Table 1:** Physical characteristics and habitual activity scores of recruited participants (*n* = 20).

Variable	Mean ± SD
Age (years)	34.8 ± 7.8
Height (cm)	177 ± 7
Weight (kg)	80.7 ± 11.7
Habitual activity	
Work Index	2.4 ± 0.6
Sport Index	3.2 ± 1.1
Vest weight (kg)	12.0 ± 1.8
Bench height (cm)	53.4 ± 3.8

## Data Availability

The data presented in this study are available on request from the corresponding author. The data are not publicly available due to ethical guidelines to protect the privacy of participants in this study.

## Supplementary Materials

The following supporting information can be downloaded at: https://www.mdpi.com/article/10.3390/nu15102316/s1, Figure S1: Relative cell counts of circulating immune cells before and after exercise in placebo and Greenshell™ mussel intervention groups; Table S1: Plasma cytokine concentrations before and at selected timepoints after the bench-stepping exercise.
