# Supplementary material for: The Efficacy of New Zealand Greenshell™ Mussel Powder Supplementation in Supporting Muscle Recovery Following Eccentric Exercise-Induced Muscle Damage in Healthy, Untrained Adult Males
Source: Nutrients. 2023 May 15;15(10):2316. doi: 10.3390/nu15102316 (PMC10221610; doi:10.3390/nu15102316)
Supplement: Supplementary file 1 [file nutrients-15-02316-s001.zip › nutrients-2382966-supplementary.pdf]

Supplementary data:

Table S1. Plasma cytokine concentrations before and at selected timepoints after the bench-stepping exercise.

	Placebo					GSM				
Cytokine	Pre	Post	24 h	48 h	72 h	Pre	Post	24 h	48 h	72 h
IL-4	4.9 ± 2.6	4.8 ± 25	4.2 ± 2.3	3.6 ± 1.9	2.8 ± 1.5	7.5 ± 4.0	7.5 ± 3.9	6.0 ± 3.2	5.2 ± 2.8	5.3 ± 2.9
IL-2	1.7 ± 0.8	1.9 ± 0.9	1.5 ± 0.7	1.6 ± 0.7	1.4 ± 0.6	2.7 ± 1.3	2.3 ± 1.1	2.2 ± 1.0	2.2 ± 0.9	1.8 ± 0.8
CXCL-10	7.5 ± 3.0	9.8 ± 3.6	8.4 ± 3.4	7.6 ± 2.8	6.8 ± 2.6	11.3 ± 4.5	12.1 ± 4.4	11.2 ± 4.5	8.1 ± 2.9	7.8 ±3.0
IL-1β	10.6 ± 4.5	14.3 ± 6.2	9.3 ± 4.0	10.0 ± 4.1	6.4 ± 2.6	11.9 ± 5.1	9.8 ± 4.2	6.7 ± 2.9	7.6 ± 3.1	7.0 ± 2.9
TNF-α	1.2 ± 0.5	1.3 ± 0.6	1.1 ± 0.5	1.0 ± 0.4	1.0 ± 0.4	1.7 ± 0.7	1.6 ± 0.7	1.3 ± 0.6	1.1 ± 0.4	1.0 ± 0.4
IL-17A	0.8 ± 0.3	0.9 ± 0.3	0.8 ± 0.3	0.7 ± 0.3	0.6 ± 0.2	1.2 ± 0.4	1.0 ± 0.4	1.0 ± 0.4	0.9 ± 0.3	0.7 ± 0.3
IL-6	2.7 ± 1.3	3.8 ± 1.8	2.5 ± 1.3	2.1 ± 0.9†	1.7 ± 0.9	5.0 ± 2.4	5.6 ± 2.7	3.3 ± 1.7	2.8 ± 1.4	2.5 ± 1.2
IL-10	1.0 ± 0.4	1.2 ± 0.5	0.8 ± 0.3	0.8 ± 0.3†	0.7 ± 0.2	1.3 ± 0.5	1.2 ± 0.5	0.9 ± 0.4	0.8 ± 0.3	0.7 ± 0.3
IFN-γ	3.1 ± 1.3	3.9 ± 1.7	2.6 ± 1.1	3.2 ± 1.2	2.3 ± 0.9	4.9 ± 2.1	4.8 ± 2.1	3.7 ± 1.6	2.8 ± 1.1	3.0 ± 1.3
IL-12p70	1.3 ± 0.6	1.7 ± 0.8	1.0 ± 0.5	1.0 ± 0.5†	0.9 ± 0.4	2.2 ± 1.0	2.1 ± 1.0	1.9 ± 0.9^	1.3 ± 0.6	1.4 ± 0.6
IL-8	1.3 ± 0.5	1.6 ± 0.6	1.3 ± 0.5	1.3 ± 0.4	1.1 ± 0.4	1.7 ± 0.6	1.7 ± 0.6	1.4 ± 0.5	1.2 ± 0.4	1.2 ± 0.4
TFG-β	1.1 ± 0.6	1.2 ± 0.8	0.8 ± 0.5	0.7 ± 0.4†	0.8 ± 0.4	1.2 ± 0.7	1.2 ± 0.7	0.9 ± 0.5	0.7 ± 0.4	0.7 ± 0.4

Cytokine concentrations in plasma samples pre-exercise, immediately post exercise and 24, 48 and 72 h after the bench-stepping exercise. Data are means ± SEM.

Abbreviations: GSM, Greenshell™ mussel; IL, interleukin; CXCL, C-X-C motif chemokine ligand; MCP, monocyte chemoattractant protein; TNF, tumour necrosis factor; IFN, interferon; TGF, transforming growth factor.

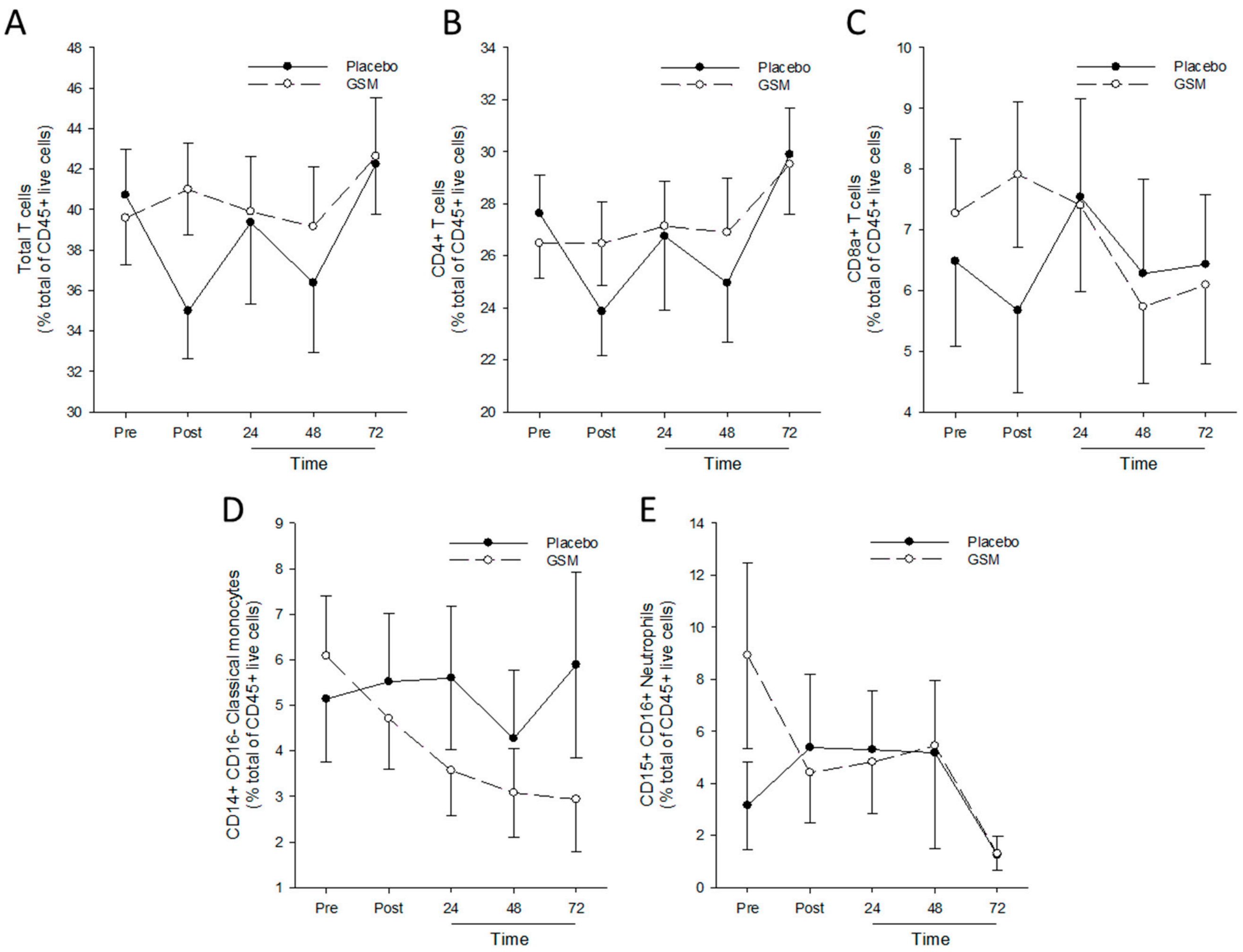

Figure S1. Relative cell counts of circulating total T cells (A), CD4+ T cells (B), CD8a+ T cells (C), CD14+ CD16- monocytes (D) and CD15+ CD16+ neutrophils (E) pre-exercise (Pre) and 0 (Post), 24, 48 and 72 h after bench-stepping exercise in placebo and Greenshell™ mussel (GSM) intervention groups. Data are means ± SEM.
